# Astrocytes Lingering at a Crossroads: Neuroprotection and Neurodegeneration in Neurocognitive Dysfunction

**DOI:** 10.7150/ijbs.109315

**Published:** 2025-04-28

**Authors:** Xiaoxia Xu, Bin Mei, Yue Yang, Junxiong Li, Juntao Weng, Yueyue Yang, Qianyun Zhu, Honghai Zhang, Xuesheng Liu

**Affiliations:** 1 Department of Anesthesiology, the First Affiliated Hospital of Anhui Medical University, Key Laboratory of Anesthesiology and Perioperative Medicine of Anhui Higher Education Institutes, Anhui Medical University, Hefei, Anhui Province, 230022, China.; 2 Department of Anesthesiology, Affiliated Hangzhou First People's Hospital, Westlake University School of Medicine, Hangzhou, 310006, China.; 3 Department of Anesthesiology, the Fourth Clinical School of Medicine, Zhejiang Chinese Medical University, Hangzhou, 310006, China.; 4 Department of Orthodontics, Affiliated Stomatological Hospital of Kunming Medical University, Yunnan Key Laboratory of Stomatology, Kunming Medical University, Kunming Yunnan 650106, China.

**Keywords:** astrocyte, neurocognitive dysfunction, epilepsy, neuroinflammation, Alzheimer's disease, postoperative cognitive dysfunction, sepsis-associated encephalopathy

## Abstract

Astrocytes, a major class of glial cells in the central nervous system, play a fundamental role in maintaining homeostasis, supporting neuronal function, and regulating synaptic activity. Recent studies have increasingly highlighted the pivotal role of astrocytes in the initiation and progression of neurocognitive dysfunction. Alterations in astrocytic morphology and functionality have been strongly associated with the onset of cognitive impairments, positioning astrocytes as key regulators in neurocognitive processes. Astrocytes influence neurocognitive function through their involvement in the uptake and release of gliotransmitters, modulation of inflammatory mediators, and metabolic regulation. These processes have been implicated in various neurodegenerative and neurocognitive disorders, including epilepsy, Alzheimer's disease, postoperative cognitive dysfunction, and sepsis-associated encephalopathy. Given the emerging role of astrocytes in these conditions, understanding the mechanisms by which they modulate neurocognitive function is essential for identifying potential therapeutic targets. This review provides an overview of the current understanding of astrocytic contributions to neurocognitive dysfunction and explores the therapeutic opportunities provided by the targeting of astrocyte-mediated pathways.

## Introduction

Due to the escalating global aging phenomenon, the number of patients with neurocognitive dysfunction caused by neurodegenerative diseases has increased dramatically^1^-^3^. Alzheimer's disease (AD), for example, is one of the leading causes of dementia worldwide and affects millions of people^4^-^6^. In addition, neurocognitive dysfunction stemming from conditions like epilepsy and sepsis has a serious impact on patients' quality of life and generates a socio-economic burden^7^-^9^. Postoperative cognitive dysfunction (POCD), which affects many elderly patients following surgery, is associated with prolonged hospitalization, long-term cognitive decline, and mortality^10^-^12^. Presently, the optimization of medical treatments to benefit such patients has become an important focus in both basic and clinical research. Astrocytes play a key role in multiple critical physiological functions such as brain homeostasis and memory^13^-^15^. This review thus summarizes current research regarding the roles of astrocytes in neurocognitive dysfunction associated with epilepsy, AD, POCD, and sepsis-associated encephalopathy (SAE) as well as the underlying mechanisms implicated therein and their potential as therapeutic targets.

## 1. Astrocyte and epilepsy

### 1.1 Mechanisms of epilepsy-induced neurocognitive dysfunction

#### 1.1.1 Amyloid and tau protein

Amyloid-β (Aβ) accumulation and tau hyperphosphorylation are central to epilepsy-associated neurocognitive dysfunction^16^-^19^. Seizures drive Aβ deposition and tau phosphorylation, potentially via stress pathways like the c-Jun N-terminal kinase (JNK), mammalian target of rapamycin (mTOR)/p70S6K, and protein kinase R -like endoplasmic reticulum kinase/eukaryotic initiation factor 2α pathways, which are activated during epileptogenesis^16^, ^20^. For example, childhood seizures correlate with midlife Aβ accumulation, suggesting long-term neurodegenerative consequences^21^, ^22^. Critically, Aβ and tau synergistically exacerbate cognitive decline: high levels of both biomarkers predict severe memory impairment, as shown in the ^11^C-Pittsburgh Compound B and ^18^F-Flortaucipir maps^23^, ^24^. Mechanistically, Aβ hyperexcites hippocampal neurons, lowering seizure thresholds, while tau potentiates network hypersynchrony and hippocampal atrophy^25^-^27^. Tau pathology also recruits cytotoxic T cells, driving neuronal loss and synaptic dysfunction^28^-^30^. Moreover, Aβ and tau protein deposition in the brain may disrupt both anatomical and functional connectivity, leading to reduced attention, language impairment, and executive dysfunction^31^, ^32^. Therapeutic strategies targeting both proteins (e.g., tau ablation) restore excitation-inhibition balance, reduce seizure severity, and improve outcomes, highlighting their intertwined roles in epileptogenic cognitive decline^33^-^35^.

#### 1.1.2 Manganese-containing superoxide dismutase (MnSOD)

MnSOD, a nuclear-encoded antioxidant metalloenzyme, primarily functions to convert O_2_ˉ to H_2_O_2_ and O_2_^36^. In pathological conditions, a single nucleotide polymorphism in the MnSOD gene results in a change from Ala to Val at the 16^th^ amino acid position of its primary structure, termed Ala16Val. This single nucleotide polymorphism is one of the most extensively studied polymorphisms in the MnSOD gene. Molecular dynamics simulation results revealed that wild-type MnSOD maintains a stable α-helical secondary structure, facilitating its translocation into mitochondria for the production of MnSOD, whereas the valine polymorphism rapidly disrupts this α-helical structure, leading to an increase in mitochondrial reactive oxygen^37^. Studies using electrophysiology and redox proteomics in cultured human pluripotent stem cell-derived cortical neurons revealed that the overproduction of mitochondrial reactive oxygen species (ROS) upregulates the expression of α-amino-3-hydroxy-5-methyl-4-isoxazole propionic acid (AMPA) and n-methyl-d-aspartate (NMDA) receptors containing glutamate receptor subunit 1 and NMDA receptor subunit 2B subunits, leading to impaired glutamate signaling, calcium overload, and excitotoxicity^38^. Inhibition of ROS reduces Ca^2+^ oscillation, mitochondrial depolarization, neuronal death, and seizure frequency in rats^39^.

**Figure 1:**
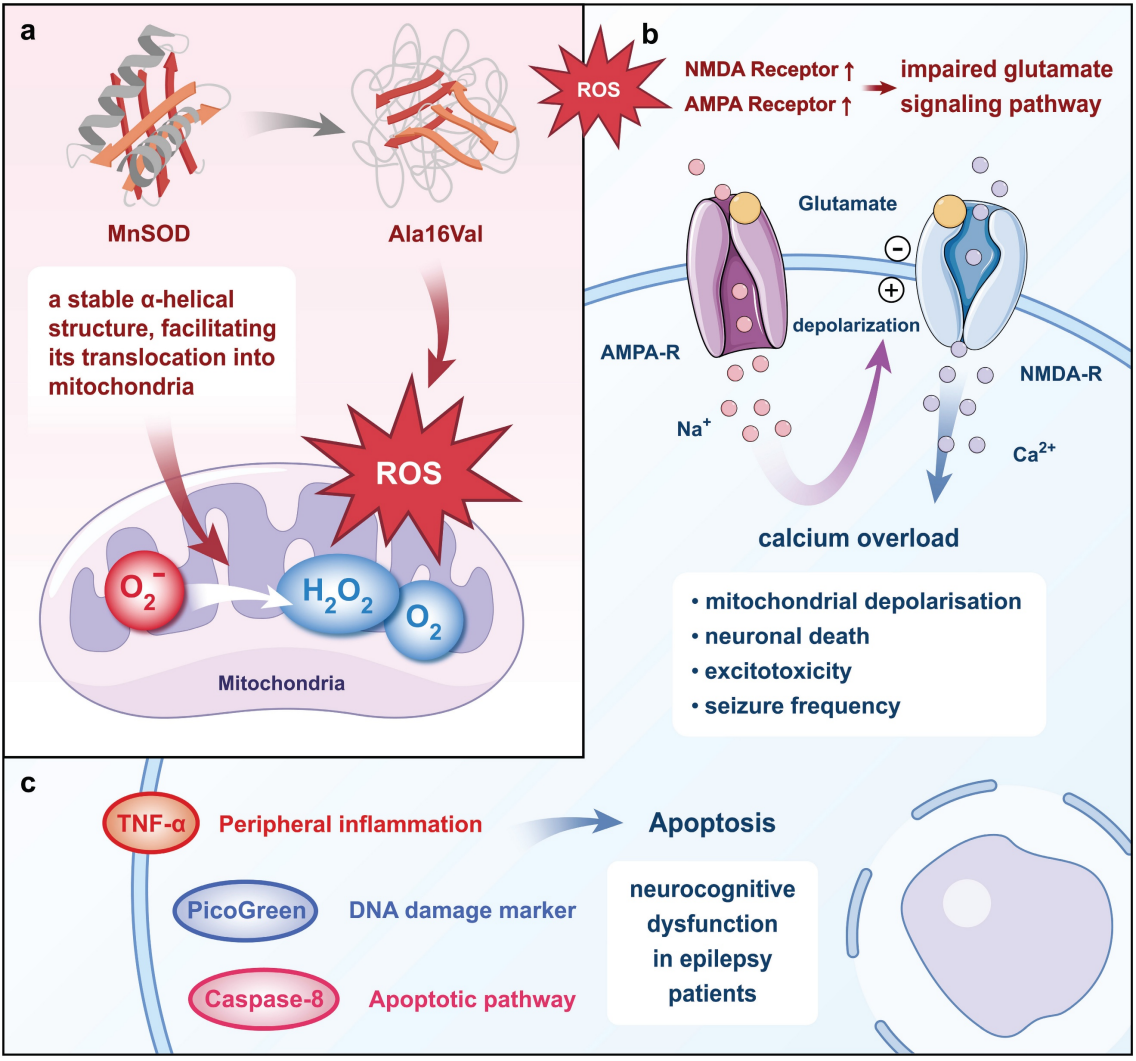
** MnSOD Ala16Val polymorphism causes neurocognitive dysfunction in epilepsy.** The Ala16Val polymorphism disrupts the α-helix structure and prevents the translocation of MnSOD precursor proteins to the mitochondria to exert antioxidant effects, leading to an increase in mitochondrial ROS. The overproduction of ROS increases the expression of the AMPA and NMDA receptors, causing calcium overload, mitochondrial depolarization, neuronal death, and an increase in seizure frequency. The Ala16Val polymorphism also induces neuronal apoptosis, DNA damage, and peripheral inflammation, ultimately leading to neurocognitive dysfunction in epilepsy.

Moreover, evidence from other studies suggested that the Ala16Val polymorphism is implicated in various processes including caspase-3-induced activation of apoptotic pathways, elevated levels of DNA damage markers (PicoGreen), and increased peripheral inflammation in epilepsy patients, all of which may contribute to neurocognitive dysfunction^40^. While the specific cell types undergoing apoptosis were not directly identified in this study, caspase-3 activation is commonly associated with neuronal and glial cell apoptosis in epilepsy^41^, ^42^. Neuronal apoptosis, in particular, is a known consequence of excitotoxicity and chronic inflammation, hallmarks of epilepsy pathophysiology^43^, ^44^. Additionally, glial cells, such as astrocytes and microglia, may also undergo apoptosis due to sustained inflammatory responses and oxidative stress, further contributing to cognitive dysfunction^45^, ^46^. The genotypes of the MnSOD Ala16Val single nucleotide polymorphism were classified as Ala/Ala, Ala/Val, and Val/Val, with the Val/Val genotype associated with higher levels of tumor necrosis factor-α (TNF-α), caspase-8, and PicoGreen^47^ (Fig. 1). Consequently, epileptic patients with the Val/Val genotype exhibit more severe inflammatory and apoptotic status^48^. Consistent with these findings, the Val/Val genotype has been closely linked to a variety of neurocognitive dysfunctions, including practice, perception, attention, language, executive function, long-term semantic memory, short-term visual memory, and total memory^47^, ^49^. Hence, the MnSOD Ala16Val polymorphism, particularly the Val/Val genotype, may play a role in the pathogenesis of epileptogenic neurocognitive dysfunction.

#### 1.1.3 Aquaporin 4 (AQP4)

AQP4 is predominantly expressed in the astrocytic endfeet around cerebral blood vessels and regulates ion and water homeostasis in the central nervous system (CNS)^50^. As a key component of the glymphatic system, AQP4 supports the brain-wide clearance of interstitial solutes, including Aβ and other neurotoxic substances^51^, ^52^. In epilepsy, AQP4 modulates the dynamics of water and ion balance in the brain, which can influence neuronal excitability and seizure susceptibility^53^, ^54^. The highly polarized expression of AQP4 water channels on astroglial endfeet is essential for efficient glymphatic function. Polarity refers to the distribution of AQP4 immunoreactivity, with higher polarity indicating a greater concentration of AQP4 in perivascular regions and lower polarity reflecting a more even distribution between perivascular endfeet and the soma^55^. It was found that the transient upregulation of AQP4 expression and depolarization following epilepsy-induced brain edema in mice may disrupt the function of the glial lymphoid system, resulting in significantly impaired performance in the Morris water maze test, which was ameliorated upon downregulation of AQP4 in Trpm4^-/-^ mice^53^. However, deficiency in AQP4 has been associated with an increase in the frequency and duration of spontaneous seizures, potentially compromising memory consolidation^54^, ^56^. Additionally, AQP4 deficiency also impairs the compensatory upregulation of glutamate transporter-1, leading to glutamate accumulation and excitatory neurotoxicity^57^. In vivo rat studies have revealed that excess glutamate can induce elevated electroencephalogram (EEG) spikes, intense epileptic discharges, epileptiform behavioral changes, and reduced neuronal counts, ultimately contributing to neurocognitive dysfunction^58^-^60^. However, blocking AQP4 in an in vitro model of 4-aminopyridine-induced epilepsy did not abolish rapid volumetric pulsations and sustained contraction of the extracellular space^61^. These findings collectively suggest that seizures may precipitate epilepsy-related memory impairment, with AQP4 potentially playing a significant role.

### 1.2 Role of astrocytes in epileptogenic neurocognitive dysfunction

Astrocytes dynamically interact with neurons by releasing signaling molecules such as ATP, glutamate, and other gliotransmitters that regulate synaptic plasticity, neuronal excitability, and network stability. Under pathological conditions, reactive astrocytes exhibit aberrant signaling, including excessive ATP release or impaired gliotransmitter uptake, which disrupts synaptic homeostasis and promotes hyperexcitability^62^, ^63^. For example, ATP released by astrocytes can activate neuronal purinergic receptors, enhance excitatory neurotransmission, and lower the seizure threshold^64^, ^65^. Conversely, glutamate uptake by astrocytes through glutamate transporter 1 (GLT-1) is essential for preventing excitotoxicity and reducing seizure susceptibility^66^. Dysregulation of these processes can lead to epileptogenesis and cognitive deficits, highlighting the importance of astrocyte-neuron crosstalk in health and disease^65^, ^67^.

Another critical function of astrocytes is their regulation of extracellular ion concentrations, particularly K⁺ and Ca²⁺. By dynamically buffering K⁺ through the inward-rectifying potassium channel Kir4.1 and spatial redistribution, astrocytes prevent extracellular K⁺ accumulation, which can depolarize neurons and lower seizure thresholds^68^. Additionally, overexpression of Kir4.1 in astrocytes attenuates presynaptic Ca²⁺ entry and release probability at excitatory synapses^69^. Similarly, astrocytes modulate extracellular Ca²⁺ levels via transporters or channels (e.g., gamma-aminobutyric acid transporter 3), maintaining ionic balance critical for synaptic transmission^70^. Impaired Kir4.1 function or dysregulated Ca²⁺ signaling disrupts ion homeostasis, lowering seizure thresholds and propagating hyperexcitability^68^, ^69^.

#### 1.2.1 Astrocytes and amyloid and tau proteins

Astrocytes play a pivotal role in regulating the metabolism of amyloid and tau proteins. In epilepsy models, astrogliosis in the prefrontal cortex and hippocampus correlates with Aβ or tau accumulation^71^, ^72^. Activated astrocytes facilitate tau propagation, as evidenced by plasma glial fibrillary acidic protein (GFAP) serving as a biomarker for Aβ positivity and a predictor of cognitive decline^73^, ^74^. GFAP has emerged as a more accurate indicator of Aβ positivity compared with other glial markers, and it has been shown to predict longitudinal deposition in whole samples, regardless of the participants' cognitive status or the presence of AD or other neurodegenerative disorders^73^. Mechanistically, the transcription factor signal transducer and activator of the transcription 3 (STAT3) pathway drives astrocyte proliferation, and its conditional knockout reduces Aβ plaques and improves spatial learning and memory decline^72^. Paradoxically, inhibiting the Janus kinase 2 (JAK2)/STAT3 pathway in reactive astrocytes does not alter tau or Aβ pathology but modulates anxiety-related behaviors, indicating pathway-specific effects^75^. This duality indicates that astrocyte activation may engage distinct molecular mechanisms—some that promote Aβ clearance (e.g., via STAT3 suppression) and others that exacerbate neuroinflammation. The unresolved interplay between astrocytic pathways and protein aggregation underscores the need for targeted therapies that selectively modulate astrocyte functions, balancing neuroprotection and inflammation mitigation to address Aβ- or tau-driven cognitive decline.

#### 1.2.2 Astrocytes and MnSOD

Astrocytes are implicated in MnSOD-mediated neurocognitive dysfunction. Zimmer et al. demonstrated that hippocampal astrocytes in patients with intractable temporal lobe epilepsy combined with hippocampal sclerosis exhibit pronounced upregulation of peroxiredoxin 6, suggesting that astrocytes play a predominant role in antioxidant gene expression in the hippocampus of epilepsy patients^76^. Critically, MnSOD deficiency in astrocytes exacerbates mitochondrial ROS accumulation, which directly contributes to epileptogenesis through multiple pathways. In vitro experiments demonstrated that exposure of human fetal astrocytes to graphene oxide induces a pro-inflammatory phenotype characterized by elevated expression of C3, exacerbating epileptic inflammatory processes and lowering seizure thresholds^76^. Downregulation of MnSOD enhances ROS production in primary astrocytes, leading to heightened cell death, whereas restoration of MnSOD levels protects astrocytes from these impairments and even reverses aggravated astrocyte damage^77^. MnSOD downregulation not only leads to elevated ROS levels but may also impair astrocytic ATP production, further destabilizing neuron-astrocyte metabolic coupling^78^, ^79^. Astrocyte injury is associated with elevated levels of S100 calcium-binding protein B and GFAP, along with decreased brain-derived neurotrophic factor (BDNF) and glial cell-derived neurotrophic factor. The levels of serum markers of astrocyte injury are correlated with insomnia severity and/or neurocognitive dysfunction^80^. Thus, MnSOD may mitigate astrocyte damage, thereby ameliorating neurocognitive dysfunction.

#### 1.2.3 Astrocytes and AQP4

Astrocytes play an important role in AQP4-mediated neurocognitive dysfunction. AQP4 expression in astrocytes exhibits a selective distribution, with a notably higher density in the endfeet membrane than in the non-endfeet membrane. Disruption of AQP4 polarization impairs astrocyte-mediated K⁺ buffering and water transport^81^, and this impaired water transport potentially causes water uptake and swelling by astrocytes^82^. Additionally, the mislocalization of AQP4 disrupts the spatial buffering of K^+^, leading to increased extracellular K^+^ levels^83^. Research has shown that AQP4 depolarization-induced dysregulation of water and K^+^ homeostasis may contribute to neurocognitive dysfunction and epileptic propensities^81^. For example, AQP4-knockout mice exhibit impaired synaptic plasticity (e.g., deficits in long-term potentiation and depression) and worsened seizure outcomes due to reduced astrocyte scar formation and dysfunctional glutamate clearance^81^, ^84^, ^85^. Astrocytes regulate glutamate via GLT-1, a glutamate transporter, and GLT-1 deficiency in zebrafish models elevates extracellular glutamate, lowering seizure thresholds^86^. Conversely, astrocytic GLT-1 achieved overexpression via the AAV-Gfa2-GLT-1-cHA vector reduces seizure duration and severity in epileptic mice by restoring glutamate homeostasis^87^.

**Figure 2:**
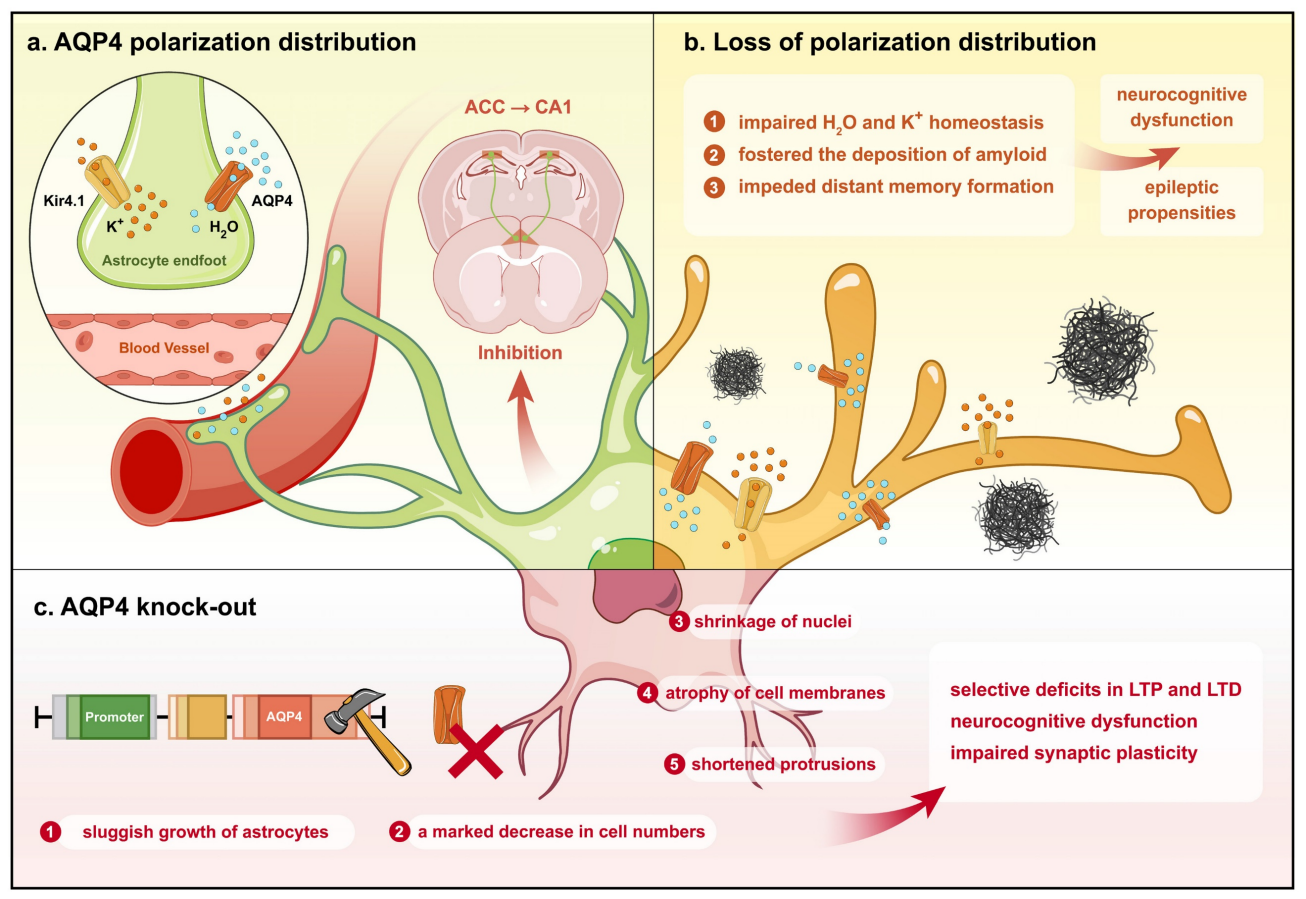
** Different conditions in which AQP4 affects neurocognition by regulating astrocyte function.** Under physiological circumstances, AQP4 exhibits a polarized distribution on astrocytes, thereby preserving physiological water and K^+^ homeostasis and restraining ACC projections to CA1 in the hippocampus for memory formation. Conversely, the depolarized distribution of AQP4 in astrocytes impairs water and K^+^ homeostasis and fosters the deposition of amyloid, inducing neurocognitive dysfunction. Knockout of AQP4 culminates in slowed astrocyte growth, markedly decreased cell numbers, shrinkage of nuclei, atrophy of cell membranes, and shortened protrusions, thereby impairing synaptic plasticity and neurocognitive function.

In vitro experiments further demonstrated that AQP4 gene silencing leads to impaired astrocyte growth with reduced cell numbers, diminished nuclei, shrunken cytosol, and shortened protrusions^88^. During memory acquisition, the anterior cingulate cortex (ACC) projects to the hippocampal CA1 region, prompting massive neuronal recruitment accompanied by activation of ACC neurons. This process is essential for distant memory formation and is inhibited by astrocytes^15^. AQP4 deficiency, then, may impede distant memory formation by impairing astrocytic function (Fig. 2). Additionally, in mice with intrahippocampal kainic acid-induced epilepsy, the neuregulin (NRG)/ErbB4 signaling pathway is upregulated in astrocytes, along with reductions in amino acid carrier 1 protein and NeuN-positive neurons, but exogenous NRG-1 treatment increases NRG/ErbB4 activity, upregulating glutamine synthetase and amino acid carrier 1 to restore glutamate homeostasis^89^.

Astrocytic pH and Ca²⁺ dynamics also modulate neuronal activity. During seizure intensification, a transient alkaline shift in astrocytes is followed by an abrupt intracellular acidic shift, which potentially stimulates gliotransmitter release and exacerbates epilepsy^90^. Given that all cellular enzymatic reactions are sensitive to Ca^2+^ and pH changes, modulation of pH and/or Ca^2+^ levels in astrocytes emerged as a potential but challenging therapeutic target for the treatment of epilepsy or the prevention of adverse plasticity associated with epileptogenesis^90^. While these intracellular changes could influence a wide range of cellular enzymes and processes, targeted approaches that selectively regulate astrocytic pH and Ca²⁺ dynamics, without broadly affecting other cell types or systemic processes, may offer a pathway to mitigate epileptogenesis with reduced off-target effects.

### 1.3 Summary

A bidirectional feed-forward relationship exists between epilepsy and amyloid and tau protein levels, wherein epilepsy triggers the accumulation of amyloid and tau proteins, and conversely, amyloid and tau proteins exacerbate seizure activity, thereby contributing to neurocognitive dysfunction. The presence of amyloid and tau proteins may indeed exacerbate neurocognitive dysfunction, potentially facilitated by astrocytic hyperplasia. Astrocytes serve as crucial intermediaries in this interplay, and amyloid deposition triggers astrocyte activation, characterized by reactive astrogliosis—a process involving morphological changes, increased expression of GFAP, and the release of pro-inflammatory cytokines. This activation of astrocytes contributes to subsequent tau protein accumulation. However, no significant impact of the astrocyte-activating JAK2/STAT pathway on amyloid and tau deposition has been observed.

Moreover, the MnSOD Ala16Val polymorphism is linked to oxidative stress, peripheral inflammation, apoptosis, and DNA damage in patients with epilepsy. These associations may explain its potential correlation with various neurocognitive dysfunctions, particularly evident in individuals with the ValVal genotype. Given that MnSOD itself exerts a protective effect on astrocytes, targeting the 16^th^ amino acid shift could potentially serve as a strategy for mitigating epilepsy-induced neurocognitive dysfunction. While inherited mutations pose challenges for direct correction, therapeutic approaches such as gene editing technologies (e.g., CRISPR-Cas9) or pharmacological modulation of downstream pathways affected by the mutation could be explored as strategies to mitigate the functional consequences of the amino acid shift. Further studies are needed to develop clinically feasible strategies for targeting this specific mutation.

Additionally, research has demonstrated that seizures impede memory consolidation and deficiency in AQP4 increases the frequency and duration of spontaneous seizures while elevating glutamate levels, triggering CNS neuroexcitotoxicity^91^. AQP4 plays a pivotal role in regulating synaptic plasticity, astrocytic growth and function, glutamate clearance, and water and K^+^ homeostasis, as well as in influencing amyloid deposition. Either knockout or silencing of AQP4 has been associated with varying degrees of neurocognitive dysfunction. Hence, targeting AQP4 may represent a viable approach for treating neurocognitive dysfunction arising from epilepsy. Therefore, astrocytes play an important role in epilepsy, and interventions aimed at ameliorating neurocognitive dysfunction through astrocyte modulation could yield favorable outcomes in epilepsy treatment.

## 2. Astrocytes and AD

### 2.1 Mechanisms of neurocognitive dysfunction in AD

#### 2.1.1 Amyloid

AD is a neurodegenerative disease associated with aging. The predominant pathological features of AD include amyloid plaques and neurofibrillary tangles in the brain^92^, ^93^. These histopathological features impair neuronal circuit function and alter the behavioral response by affecting the activation of neurotransmitter receptors^94^, ^95^. In primary neural cultures, exposure to Aβ was shown to result in a 33% decrease in cell survival and a 38% reduction in neurite extensions among surviving cortical neurons^73^. Furthermore, studies have demonstrated that elevated levels of Aβ, amyloid precursor protein (APP), or APP C-terminal fragments increase the expression of adenosine A2A receptors in the hippocampal and neocortical astrocytes of aging mice. This A2A receptor up-regulation may contribute to memory impairment in patients with AD^96^.

Then how does Aβ contribute to neurocognitive dysfunction? A study comparing levels of protein kinase C alpha (PKCα) protein in the frontal cortex of post-mortem human brains from individuals with AD and age-matched controls via immunoblotting revealed upregulation of PKCα in AD brains^97^. To investigate whether enhanced PKCα signaling output was associated with AD, the researchers studied mice harboring the PKCα M489V mutation, which is associated with increased constitutive PKCα activity. In these mice, the mutation was found to alter the brain phosphoproteome, reduce the spine density in hippocampal neurons, enhance Aβ-induced synaptic inhibition, and ultimately impair spatial learning memory in the Barnes maze test without affecting Aβ levels in the brain. These findings suggest that Aβ may inhibit synapses by enhancing PKCα activity, leading to cognitive decline^97^. Triggering receptor expressed on myeloid cells 2 (TREM2), a receptor on microglia, plays a critical role in regulating microglial migration and phagocytosis of oligomeric Aβ and amyloid plaques^98^-^100^. In a study using the 5XFAD mouse model of AD, TREM2 was activated by a TREM2 agonistic antibody tetra-variable domain immuno-globulin (Ab18 TVD-Ig), which increased TREM2 activation by 100-fold compared to its bivalent form. This enhanced activation significantly reduced amyloid burden, increased microglial migration toward and phagocytosis of amyloid plaques, and decreased the presence of lysosome-associated membrane protein 1, a marker of dystrophic neurites surrounding plaques^101^. Additionally, the treatment enhanced the expression of synaptophysin (a presynaptic marker) and postsynaptic markers in the hippocampus and cortex, indicating improved synaptic function. Behavioral tests revealed that the treatment reduced anxiety-like behaviors in the open arms of the elevated plus maze test and improved hippocampal-dependent contextual memory during the fear conditioning test, indicating enhanced cognitive function^101^.

Are reductions in Aβ content protective against neurocognitive dysfunction? A study using a mouse model that altered GPR3 (an orphan G protein-coupled receptor implicated in AD) to be G protein-biased (engineered to favor G protein signaling over β-arrestin pathways by mutation of phosphorylation sites in its C-terminus) demonstrated that this change led to reduced endogenous Aβ40 and Aβ42 production and decreased amyloid plaque area^102^. Reducing amyloid deposition has been shown to reverse memory deficits and anxiety symptoms, thereby improving cognitive performance in mice^103^, ^104^. However, reducing the accumulation of amyloidogenic APP C-terminal fragments, soluble Aβ, Aβ oligomers, and amyloid deposits was not found to ameliorate neural network dysfunction and behavioral abnormalities^105^. Thus, amyloid deposition is implicated in a myriad of neurocognitive deficits, and its removal may ameliorate only some aspects of AD neuropathology.

#### 2.1.2 Tau protein

The accumulation of tau protein tangles represents a pivotal component of AD-related neurocognitive dysfunction^106^. Levels of plasma biomarkers, including GFAP, t-tau, p-tau181, and p-tau231 are elevated in patients with preclinical AD, with GFAP and p-tau181 increasing over time. These observations emphasize the diagnostic and longitudinal monitoring potential of plasma GFAP and p-tau isoforms in preclinical AD^107^. From comparisons of various plasma biomarkers in early-stage AD cohorts, longitudinal increases in p-tau217 are specifically linked to lower Mini-Mental State Examination scores and brain atrophy, and in a delayed recall memory test, only the slope of p-tau217 is significantly associated with cognitive decline^108^. Symptomatic younger AD patients show stronger tau positron emission tomography signals in the frontoparietal hub, a region critical for AD-related cognition, which is highly connected to other brain regions. These independently verified findings suggest that symptomatic episodes in younger AD patients correlate with heightened tau pathology in the brain hub, accelerating tau propagation across interconnected brain regions and cognitive decline^109^. Plasma levels of p-tau217 increase in early preclinical AD, preceding tau-positron emission tomography positivity^110^.

In the brains of AD patients, pathological tau protein accumulation leads to neuronal loss, synaptic dysfunction, and subsequent neurocognitive dysfunction^92^, ^111^. *BIN1*, a susceptibility gene for AD, has multiple isoforms, with 10 isoforms identified as being expressed in the CNS. The peptide encoded by exon 7 of the *BIN1* N-BAR structural domain exhibits a significant inverse association with AD-related traits, particularly tau protein tangle accumulation, and reduced expression of the *BIN1* isoform containing exon 7 correlates with increased tau protein tangle deposition and subsequent neurocognitive dysfunction^112^. Aβ facilitates the accumulation of pathogenic tau, while TREM2 can slow AD progression and reduce tau-driven neurodegeneration by limiting this process^113^. Genome-wide miRNA screening in Drosophila identified inhibitors of tau-mediated AD pathology, pinpointing the miR-9 family as potent inhibitors of human tau overexpression, and overexpression of *CG11070*, a target gene of Drosophila miR-9a, or its mammalian homolog *UBE4B*, increased tau ubiquitination and degradation, leading to reduced total and phosphorylated tau levels, thereby suggesting a potential innovative therapeutic strategy for AD^114^. Thus, the tau protein tangles play an important role in AD-induced neurocognitive dysfunction.

#### 2.1.3 P2Y1 purinoreceptor (P2Y1R)

**Figure 3:**
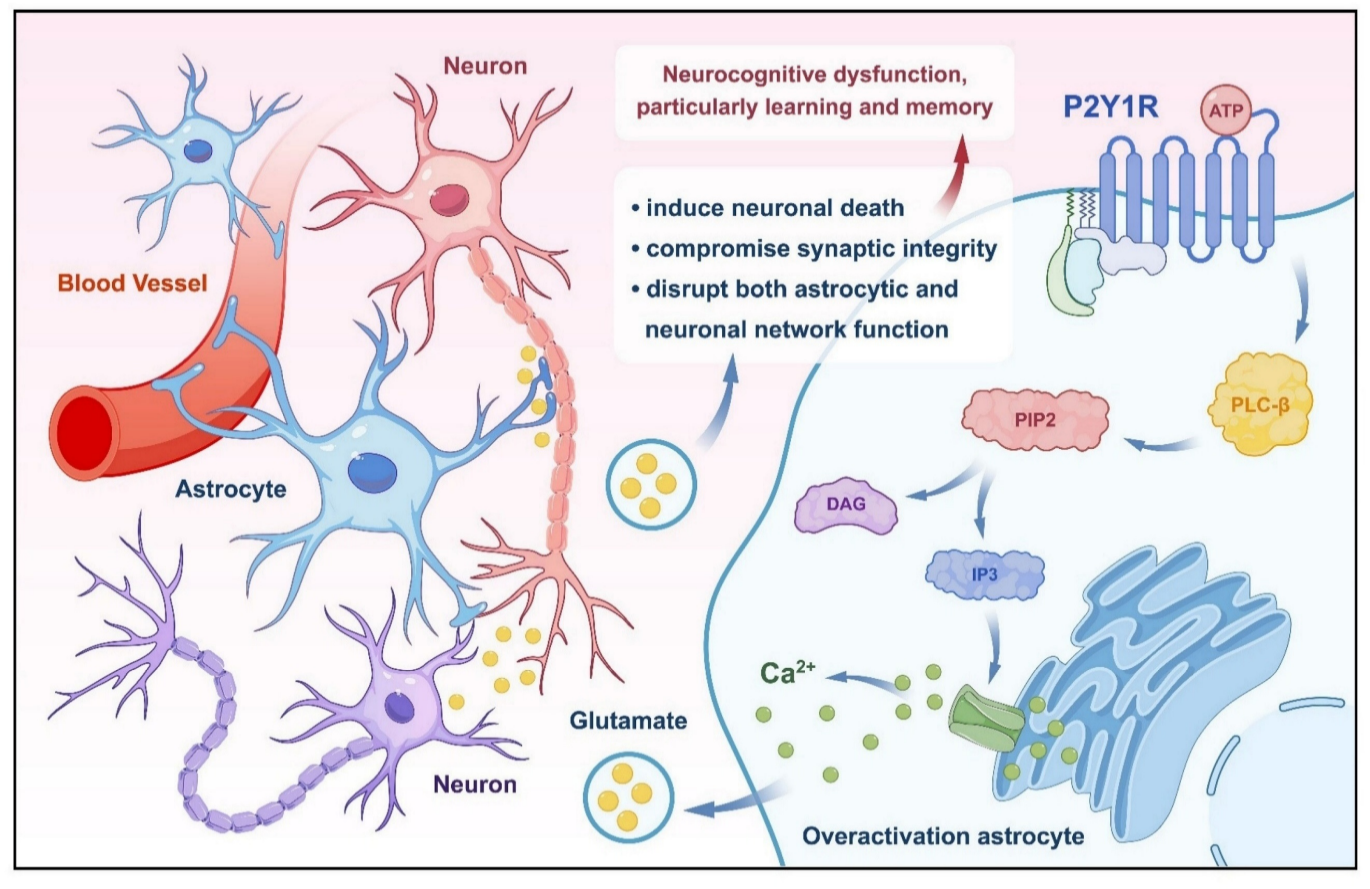
** P2Y1R improves neurocognitive function by regulating glutamate release from astrocytes.** Activation of P2Y1R by ATP leads to overactivation of astrocytes, triggering the release of glutamate. Excess glutamate levels induce neuronal death, compromise synaptic integrity, and disrupt both astrocytic and neuronal network function. These pathological processes collectively contribute to neurocognitive dysfunction, particularly impairing learning and memory.

The P2YR purinoceptors, including P2Y1R, are metabotropic ATP receptors. In the context of AD, P2Y1R plays a significant role in neurocognitive dysfunction. Research indicates that excessive activation of astrocytes is an important factor in neuronal-glial network dysfunction in AD, with P2Y1R-mediated signaling pathways implicated in driving astrocyte overactivation^115^. Specifically, selective activation of P2Y1R in cultured astrocytes has been shown to induce massive glutamate release, a phenomenon associated with neuronal death in the rat hippocampus^116^, ^117^. Activation of P2Y1R in the medial prefrontal cortex disrupts the inhibitory control and behavioral flexibility mediated by increased mesocorticolimbic activity and local disinhibition, suggesting a role for P2Y1R in modulating cognitive-behavioral responses in the prefrontal cortex^118^. Furthermore, extracellular ATP regulates neuronal damage and memory impairment primarily through activation of P2Y1R, and antagonizing P2Y1R protects against memory deficits^119^. Restoration of inhibitory homeostasis, a process crucial for resilience to Aβ accumulation, structural preservation, and synaptic hyperinhibition, can be achieved by blocking P2Y1R in calreticulin interneurons, which is selectively dysfunctional during AD pathogenesis^120^ (Fig. 3). Hence, antagonizing P2Y1R presents a potential therapeutic approach for ameliorating the neurocognitive dysfunction caused by AD.

### 2.2 Role of astrocytes in AD-induced neurocognitive dysfunction

#### 2.2.1 Astrocytes and amyloid

In the context of AD, a reciprocal regulatory relationship exists between astrocytes and Aβ. Neuroinflammation, driven by reactive astrocytes and microglia, facilitates the development of neurodegenerative diseases and cognitive decline^121^-^123^. Astrocytes transition from a homeostatic state to a reactive state in response to Aβ aggregates, releasing pro-inflammatory cytokines, chemokines, and synaptotoxic secretions^124^, ^125^. This inflammatory environment perpetuates neuronal damage, disrupted synaptic plasticity, and accelerated Aβ aggregation, forming a vicious cycle that drives disease progression^126^. For example, Aβ plaques activate astrocytes via Toll-like receptor 4, triggering nuclear factor- kappa B (NF-κB)-dependent transcription of pro-inflammatory mediators^127^. These reactive astrocytes upregulate Na^+^/H^+^ exchanger isoform 1, enhancing Aβ accumulation^128^.

Despite this detrimental feedback loop, astrocytes also exhibit protective roles in modulating Aβ pathology^129^. Astrocytes differentiate into two distinct C3-positive and C3-negative reactive populations, in which soluble adenylyl cyclase and compartmented, nuclear- and cytoplasmic-localized cyclic adenosine monophosphate in reactive astrocytes act as a molecular switch for neuroprotective astrocyte reactivity^130^. In neurodegenerative diseases like AD, an imbalance between neurotoxic and neuroprotective astrocytes has been observed in animal models and human patients. For example, in a mouse model of neurodegeneration, neuroprotective astrocytes initially emerge in an intermediate state but then shift to a neurotoxic phenotype under sustained neuroinflammation, and targeting mTOR in astrocytes can alleviate astrocyte neurotoxicity^131^.

Alterations in amyloid pathology are accompanied by the activation and hypertrophy of microglia and astrocytes, which may limit Aβ progression under specific conditions. While the role of glial activation in AD is complex and context-dependent, one study found that the observed glial response appears to be protective, as these glial changes could limit the progression of amyloid pathology in G protein-biased GPR3 AD mice^102^. Mechanistically, astrocytes are recruited to Aβ plaques and enhance autophagosome generation, reducing soluble and insoluble Aβ levels in hippocampal and cortical tissues^96^. This process may involve signaling pathways such as the phosphatidylinositide 3-kinase/protein kinase B/actin, Sirtuin 1/AMP-activated protein kinase/sterol response element binding protein 2, and chemerin/chemokine-like receptor 1/stimulator and deactivation of ephrin receptor A4/c-Abl pathways^132^, ^133^. Furthermore, astrocytes regulate Aβ clearance through cholesterol homeostasis: maintaining low neuronal cholesterol levels inhibits Aβ accumulation, while astrocytic dysfunction impairs Aβ clearance via NF-κB inflammatory signaling^124^, ^134^.

Astrocytes also secrete clusterin, a synaptogenic factor with anti-amyloid properties, which enhances synaptic transmission and reduces Aβ levels^135^, ^136^. Conversely, Aβ itself can subvert these protective mechanisms. For instance, Aβ downregulates phagocytic receptors (e.g., Mertk, Megf10) in astrocytes, directly impairing their ability to clear oligomeric Aβ and encapsulate dystrophic synapses near plaques^137^. This bidirectional interaction underscores the duality of astrocyte function: while they possess intrinsic capacities to mitigate Aβ pathology (e.g., via autophagy, clusterin secretion), chronic Aβ exposure disrupts these mechanisms, exacerbating neuroinflammation and neurodegeneration.

#### 2.2.2 Astrocytes and apolipoprotein E (ApoE)

Astrocytes regulate tau protein aggregation and phosphorylation through the influence of ApoE4, the mutation of which is currently the strongest genetic risk factor associated with late-onset AD. Notably, tau protein accumulation has been observed in astrocytes within the dentate gyrus of AD patients. In mice, overexpression of 3R tau protein (contains three microtubule-binding repeats in the carboxy-terminal) in dentate gyrus astrocytes alters mitochondrial dynamic and function, resulting in impaired spatial memory capacity^138^. ApoEε4, one of the high-frequency alleles of ApoE4 and a major genetic risk factor for AD, enhanced tau protein aggregation and phosphorylation in neurons when expressed in astrocytes^139^. In vivo and in vitro investigations have demonstrated that phosphatidylinositol proteoglycan 4, secreted by astrocytes, strongly interacts with APOE4 and exacerbates APOE4-induced diffusion and hyperphosphorylation of tau^140^. Utilizing brain organoid models derived from induced pluripotent stem cells, a study revealed that homozygous conversion of ApoE4 to ApoE3 attenuates the ApoE4-associated phenotype in brain organoid cells from AD patients^72^. Removal of astrocyte ApoE4 alleviates tau-induced synaptic deficits and reduces phagocytosis of synaptic components by microglia^141^. Hippocampal synaptic dysfunction and dendritic deficits associated with microglial activation may underlie memory deficits^142^. These findings collectively indicate that ApoE4 may drive memory deficits in AD through its effects on astrocytes. APOE4 disrupts the neuronal microenvironment by impairing the cholesterol metabolism balance in astrocytes, activating inflammatory signaling pathways, and causing dysregulation of extracellular matrix production. These disruptions lead to synaptic dysfunction and reduce the efficiency of neural networks, ultimately contributing to AD-related memory deficits^143^. Specifically, APOE4-induced cholesterol dysregulation in astrocytes inhibits the myelination of oligodendrocytes, further exacerbating neuronal dysfunction and impairing memory formation^144^. Mechanistically, astrocytes regulate tau accumulation through the histone deacetylase 7/transcriptional factor EB lysosomal and NF-κB/chemokine (C-C motif ligand 2, C-X-C motif ligand 1, and interleukin [IL]-6) signaling pathways^145^, ^146^. Thus, APOE4 exerts its detrimental effects on memory by causing a combination of cholesterol metabolism disruption, inflammation, and synaptic dysfunction, highlighting the central role of astrocytes in AD pathogenesis.

#### 2.2.3 Astrocytes and P2Y1R

P2Y1R exerts significant effects on astrocytes, particularly in the context of AD. Research has shown that long-term intracerebroventricular infusion of P2Y1R inhibitors, monitored through the vivo two-photon microscopy, preserves normal astrocytic and neuronal network function, bolsters synaptic structure integrity, and safeguards hippocampal long-term potentiation (LTP). In the early stages of AD, P2Y1R mediates a prolonged duration of the response to ATP-induced [Ca^2+^]_i_^147^. Notably, inhibition of astrocytic Ca^2+^-dependent overexcitatory purinergic signaling, primarily mediated by P2Y1R, rescues theta burst-induced LTP, suggesting the critical role of spontaneous astrocytic Ca^2+^ signaling in maintaining CA3-CA1 synaptic plasticity^148^. Long-term administration of P2Y1R antagonists in AD mice, or the genetic deletion of astrocyte-specific P2Y1R downstream signaling pathways, prevents the decline in spatial learning and memory in these mouse models^115^. Therefore, activation of P2Y1R in astrocytes may be an important mechanism underlying the neurocognitive dysfunction in AD.

### 2.3 Summary

In summary, Aβ deposition leads to impaired neuronal circuit function and altered behavioral response. Clearance of amyloid improves memory deficits, anxiety symptoms, and cognitive performance, although it does not address network dysfunction and behavioral abnormalities. Astrocytes are able to clear Aβ via autophagy, but Aβ itself impairs the ability of astrocytes to clear Aβ by downregulating the expression of astrocyte phagocytic receptors. Augmentation of clusterin levels in astrocytes has shown promise for reducing Aβ levels and ameliorating the associated neurocognitive dysfunction.

Accumulation of tau protein also causes cognitive and memory impairments. Astrocytic expression of ApoE4 exacerbates tau protein accumulation, and removal of astrocytic ApoE4 appears to mitigate synaptic loss and phagocytosis, potentially attenuating memory deficits. Activation of P2Y1R not only triggers glutamate release, leading to hippocampal neuronal death, but also disrupts the inhibitory control and behavioral flexibility mediated via increased mesocortical limbic activity and local disinhibition, ultimately leading to neuronal damage and memory impairment. Conversely, inhibition of P2Y1R normalizes astrocytic and neuronal network function, enhances synaptic structure integrity, and preserves hippocampal LTP. In conclusion, astrocytes play an important role in AD, and interventions targeting astrocytic function hold promise for improving AD-related neurocognitive dysfunction, thus bearing important implications for clinical treatment strategies.

## 3. Astrocytes and POCD

### 3.1 Mechanism of POCD

#### 3.1.1 Connexin-43 (Cx43)

Cx43 expression may be involved in the progression of POCD. Sevoflurane, a volatile anaesthetic, increases the expression of Cx43 and induces neuronal apoptosis by activating the JNK signaling pathway in the rat hippocampus on postnatal day 7, which can lead to neurocognitive dysfunction during puberty^149^. Cx43 is the predominant gap junction (GJ) protein in the nervous system, serving vital physiological functions including regulation of ion transport, regulation of substrate exchange, and facilitation of intercellular signaling^150^, ^151^. A study investigating long-term isoflurane-induced perioperative neurocognitive dysfunction showed that prolonged exposure to isoflurane results in the uncoupling of Cx43-based GJs without altering the total expression of Cx43, which increases oxidative stress and neuroinflammation in the hippocampus and primary astrocytes, leading to neurocognitive dysfunction, as evidenced by the results of Y-maze and fear conditioning tests, while increased Cx43-based GJ formation ameliorates these effects^152^. Notably, the Cx43 protein can form both GJ channels and hemichannels^153^. After long-term isoflurane anesthesia, enhancement of the total Cx43 hemichannels increases astrocyte permeability, neuronal excitability, and neuronal toxicity^154^. Research has indicated that internal fixation of tibial fractures induces the opening of astrocytic Cx43 hemichannels, triggering an inflammatory response in the hippocampus and resulting in neurocognitive dysfunction in aged mice^155^. These findings suggest that Cx43 contributes to POCD not only in surgical procedures (e.g., orthopedic surgery) but also in models of anesthesia induction (e.g., prolonged exposure to isoflurane or sevoflurane). The dual role of Cx43 (through gastrojunctional uncoupling and hemichannel activation) highlights its widespread involvement in neuroinflammation and synaptic disruption in response to various perioperative stressors, further supporting its potential as a mechanistic target in the pathogenesis of POCD.

#### 3.1.2 MicroRNA-146a (miR-146a)

MiR-146a is a small non-coding RNA that plays a key role in the negative regulation of the innate immune response. MiR-146a dysregulation is associated with a variety of inflammatory diseases^156^, ^157^. MiR-146a may negatively regulate surgery-induced hippocampal neuroinflammation and ameliorate cognitive decline through inhibition of the IL receptor associated kinase 1 (IRAK1)/tumor necrosis factor receptor associated factor 6/NF-κB pathway. Thus, overexpression of miR-146a attenuates early postoperative neurocognitive dysfunction by suppressing surgery-induced hippocampal neuroinflammation in mice. Downregulation of miR-146a may exacerbate hippocampal-dependent learning memory deficits and hippocampal inflammation in POCD mice^158^. A study on the neuroinflammatory response mediated by *Escherichia coli* infection suggested that inhibition of *Escherichia coli*-induced miR-146a in vivo may further promote the production of inflammatory cytokines, exacerbate astrocyte and microglial reactivity, and shorten the survival time of mice via the toll-like receptor/NF-κB or epidermal growth factor receptor/NF-κB signaling pathways^159^. A reduction in lysosomal activity (Lyso Tracker and *BECN1* gene) and enhanced expression of synaptic/axonal transport-related genes (*DLG4*, *KIF5B*, and *DYNC1H1*) in motor neurons occurs after co-culture with astrocytes with altered miR-146a expression^160^. In addition, research showed that expression of *B-cell translocation gene 2* (*BTG2*), a tumor suppressor gene^161^, is increased in a mouse model of POCD, and inhibition of *BTG2* reduces neuronal apoptosis, necrosis, and brain damage, thereby attenuating neurocognitive dysfunction. MiR-146a negatively regulates the expression of *BTG2*^162^, suggesting that increasing the level of miR-146a could potentially prevent and treat POCD by inhibiting *BTG2*.

#### 3.1.3 Abnormal activation of N-methyl-D-aspartate receptor (NMDAR)

Aberrant activation of NMDAR may be involved in the onset and development of POCD. The BDNF/tropomyosin-related kinase receptor B (TrkB) signaling pathway is considered an important mechanism in POCD. BDNF is known to activate the high-affinity TrkB, including the full-length (TrkB-FL) and truncated (TrkB-T) isoforms^163^. Anesthesia- and surgery-induced neuroinflammation excessively activates NMDAR, which in turn triggers excessive activation of calpain, causing TrkB-FL truncation, dysregulation of the BDNF/TrkB signaling pathway, loss of dendritic spines, and apoptosis, resulting in neurocognitive dysfunction in aged mice. Notably, treatment with the NMDAR antagonist memantine or the calpain inhibitor MDL-28170 can attenuate these abnormalities, suggesting that inhibition of abnormal activation of NMDAR or TrkB-FL truncation may be a strategy for treating POCD^11^.

Memantine is commonly used in the treatment of AD. Experiments in POCD mice have shown a potential protective effect of memantine on memory, depression-like behavior, and social novelty preferences^164^. Isoflurane, another volatile anesthetic, may induce reversible neurocognitive dysfunction, and sustained upregulation of the extra-synaptic NMDAR 2B subunit after isoflurane anesthesia may suppress synaptic transmission and thus contribute to neurocognitive dysfunction^165^. Therefore, upregulation of the extra-synaptic NMDAR 2B subunit could be another potential mechanism of POCD induction by NMDAR overactivation.

### 3.2 Role of astrocytes in POCD

#### 3.2.1 Astrocytes and Cx43

Cx43 plays an important regulatory role in astrocytes. Astrocytes form astrocyte networks through connections of GJs, which are mainly composed of Cx43, forming astrocyte networks^166^-^168^. In long-term isoflurane anesthesia-induced POCD, a significant reduction in Cx43 formation occurs in the hippocampus and primary mouse astrocytes along with impaired function of the astrocyte network and elevated levels of IL-1β and IL-6 in the hippocampus and astrocytes, and the mice exhibit severe neurocognitive dysfunction^169^. Deletion of Cx30 and Cx43 impairs astrocyte GJ coupling, leading to activation of astrocytes, enhancement of excitability and excitatory synaptic transmission in hippocampal CA1 neurons, and reduced LTP, ultimately resulting in deficits in sensorimotor performance and spatial learning and memory^170^. Abnormal over-opening of Cx43 hemichannels on reactive astrocytes exacerbates inflammatory response and cell death in CNS pathology^171^. Cx43-specific deletion in astrocytes improves neurocognitive dysfunction by reducing astrocyte proliferation and increasing synaptic function^172^. Moreover, Cx43 deletion before and after birth did not impair the laminar distribution of neocortical excitatory neurons. Consequently, mice deficient in Cx43 during the early postnatal period show normal anxiety-like behavior, depression-related behavior, and learning and memory-related behavior in adolescence^173^. Therefore, inhibition of Cx43 hemichannels in astrocytes may be a potential target for the treatment of POCD.

#### 3.2.2 Astrocytes and miR-146a

**Figure 4:**
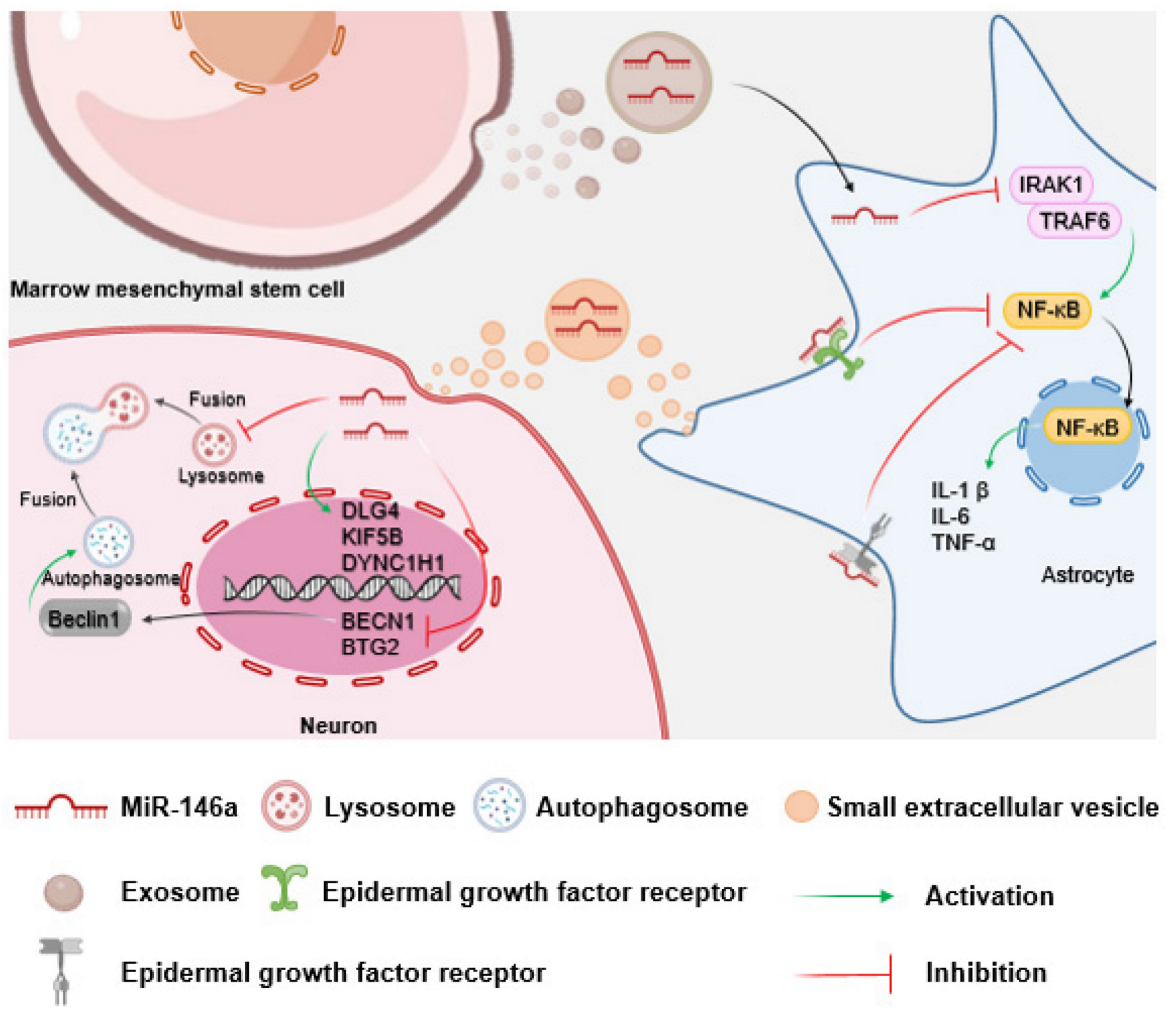
** Schematic of the impact of miR-146a on neurons mediated by astrocytes.** Bone marrow mesenchymal stem cells secrete miR-146a-containing exosomes to inhibit neuroinflammation and protect neurocognitive function by suppressing the IRAK1/TRAF6/NF-κB pathway in astrocytes. Furthermore, astrocytes can regulate neuronal autophagy and axonal transport function through the paracrine pathway.

MiR-146a exhibits critical functions in the modulation of astrocyte function. In vitro experiments have shown that exosomal miR-146a secreted by bone marrow mesenchymal stem cells is taken up by astrocytes, where it suppresses NF-κB signaling and inflammation^174^. This aligns with the view that miR-146a is a negative feedback regulator of the astrocyte-mediated inflammatory response^175^. Therefore, astrocyte-specific miR-146a overexpression may attenuate neuroinflammation, ameliorate neuritis pathology, and prevent neuronal loss, thereby reducing neurocognitive dysfunction in learning and memory^176^, ^177^. Given that astrocytes play a pivotal role in synapse formation, restoration of astrocyte function could facilitate the correction of aberrant synapse formation and improve neurocognitive dysfunction^174^. Thus, miR-146a may reduce neuroinflammation by regulating astrocyte function, thereby improving neurocognitive dysfunction (Fig. 4).

#### 3.2.3 Astrocytes and NMDAR

Neurons and astrocytes regulate each other reciprocally via neuronal NMDAR. Evidence suggests an inter-cellular signaling mechanism between neurons and astrocytes, wherein neuronal NMDAR activation promotes neuroglial cell divisions during postnatal development or as a major component of neuroinflammation after mild brain injury^178^. Astrocytes release D-serine to stimulate NMDAR, causing glutamatergic synapses to undergo short-term enhancement and promoting water-seeking memory and water intake^179^. Hippocampal astrocytes modulate NMDAR via the glutamate-permeable anion channel Bestrophin1-mediated co-release of D-serine and glutamate, fostering heterosynaptic long-term depression and metaplasticity, thereby contributing to cognitive flexibility^180^. However, under pathological conditions, hyperactivated astrocytes release excessive levels of excitatory transmitters such as glutamate and D-serine, inducing overactivation of the NMDAR in neurons, calcium overload, and ultimately apoptotic neuronal death due to excitotoxicity^181^, ^182^. Mechanistically, astrocytic dysfunction contributes to POCD by impairing synaptic plasticity via the Sirtuin 1/BDNF and BDNF/tropomyosin receptor kinase B signaling pathways^183^-^185^. L-serine, a precursor of D-serine, acts as a co-agonist of synaptic NMDAR and is required for synaptic plasticity^186^, ^187^. Dietary supplementation with L-serine can prevent synaptic and behavioral deficits in mice^188^. Therefore, avoiding excessive activation of NMDAR caused by astrocyte hyperexcitability may be beneficial in preventing and treating POCD.

### 3.3 Summary

Both surgery and anesthesia can contribute to POCD^189^. Cx43 forms GJ channels and hemichannels. Uncoupling of Cx43-based GJs increases oxidative stress and neuroinflammation levels, leading to neurocognitive dysfunction, while increased Cx43-based GJs coupling ameliorates these effects. Elevation of Cx43 hemichannels contributes to neurocognitive dysfunction by promoting the inflammatory response, and inhibition of Cx43 expression improves learning and memory function. Astrocyte-specific deletion of Cx43 improves neurocognitive dysfunction by reducing astroglial proliferation and increasing synaptic function. At the molecular level, miR-146a blunts hippocampal neuroinflammation to minimize neurocognitive dysfunction. In addition, miR-146a inhibits neuronal apoptosis, necrosis, and brain damage by negatively regulating *BTG2* expression, ultimately improving neurocognitive dysfunction. Increased miR-146a expression in astrocytes reduces neuroinflammation and thus improves neurocognitive dysfunction. Overactivation of NMDAR and sustained upregulation of the extrasynaptic NMDAR 2B subunit can lead to neurocognitive dysfunction. Under pathological conditions, hyperactive astrocytes release excessive amounts of excitatory transmitters, resulting in neuronal apoptosis due to excitotoxicity. Thus, astrocytes play a crucial role in POCD, and modulation of astrocytes may be an important approach in improving POCD-induced neurocognitive dysfunction.

## 4. Astrocytes and SAE

### 4.1 Mechanisms of neurocognitive dysfunction caused by SAE

#### 4.1.1 Nucleotide-binding oligomerization domain (NOD) like receptor protein 3/cysteine aspartate protease 1 (NLRP3/caspase-1)

NLRP3 and caspase-1, inflammation-associated proteins, are pivotal players in the pathogenesis of SAE and contribute significantly to neurocognitive dysfunction. Studies in cecal ligation and puncture (CLP)-induced models demonstrated hippocampal-dependent memory deficits coinciding with elevated NLRP3- and caspase-1-positive neurons in the hippocampal CA1 region as well as increased hippocampal contents of NLRP3/caspase-1 signaling, gasdermin-D, and pro-inflammatory cytokines within the hippocampus, suggesting that the NLRP3/caspase-1 signaling pathway may play an important role in neuronal apoptosis and the resultant neurocognitive dysfunction during SAE progression^190^. Moreover, inhibition of NLRP3 or caspase-1 was shown to reverse neurobehavioral abnormalities^190^, ^191^. The NLRP-caspase-1 complex was shown to direct the localization of caspase-1 activity^192^, while inhibition of NLRP3 mitigates microglia activation and neurocognitive dysfunction, thereby attenuating CLP-induced neurocognitive dysfunction^193^. Similarly, inhibition of caspase-1 can prevent neurocognitive dysfunction by reducing expression of IL-1β, monocyte chemotactic protein-1, and TNF-α in serum and brain tissue in septic mice, resulting in the inhibition of microglial activation, reduced blood-brain barrier (BBB) disruption and ultrastructural damage to brain tissue, protection of synaptic plasticity, and preservation of LTP^194^. Behavioral assessments in mouse models of lipopolysaccharide (LPS)-induced SAE, utilizing novel object recognition, elevated plus maze, and tail suspension tests, highlighted the efficacy of pharmacologically inhibiting the NLRP3 inflammasome for alleviating cognitive deficits, mood disturbances, inflammation, and BBB dysfunction in SAE^195^. Specifically, inhibition of NLRP3/caspase-1 signaling with NU9056 inhibits the classical pathway of gasdermin-D-mediated pyroptosis and attenuates BBB injury, further ameliorating SAE pathology^196^. Moreover, inhibition of NLRP3/caspase-1/toll-like receptor 4 signaling attenuates neuroinflammation, NLRP3 inflammasome activation, neuronal damage, and mitochondrial damage in young rats^197^. These findings underscore the therapeutic potential of targeting the NLRP3/caspase-1 pathway as an approach to mitigate the neurocognitive dysfunction associated with SAE.

#### 4.1.2 Neuronal damage

Neuronal damage is the primary driver of SAE-associated neurocognitive dysfunction. In rodent models, SAE induces hippocampal iron overload, activating apoptotic pathways linked to neurocognitive deficits^198^. Iron chelation with deferoxamine mitigates neuronal apoptosis by reducing iron deposition and activating the nuclear factor E2-related factor 2 (Nrf2)/glutathione peroxidase 4 antioxidant pathway, thereby preserving synaptic integrity and improving cognitive outcomes^199^. Electroacupuncture further confers neuroprotection by ameliorating neuronal damage, synaptic injury, and oxidative stress through Nrf-2/heme oxygenase-1 (HO-1) signaling^200^. Additionally, CLP and low-dose isoflurane (0.7%) increase hippocampal HO-1, reducing neuronal injury and SAE severity^201^. These findings highlight how oxidative stress and ferroptosis offer critical targets for intervention, emphasizing the potential of combining antioxidant and iron-chelating therapies to improve neurocognitive recovery in sepsis survivors.

#### 4.1.3 BBB damage

BBB damage is another critical mechanism underlying SAE. Sepsis induces substantial systemic inflammation, neuroinflammation, BBB leakage, and neurocognitive dysfunctions^205^. Exposure of mice to LPS activated mitochondrial localization of dynamin-related protein 1, provoking oxidative stress, upregulating the expression of vascular permeability modulators, and inducing mitochondrial defects in primary microvascular endothelial cells, consequently heightening BBB permeability^202^. However, inhalation of 2% H_2_ was found to reduce BBB permeability through Nrf2-induced gene expression, thereby alleviating SAE-associated neurocognitive dysfunction^203^. Moreover, subcutaneous injection of recombinant human brain natriuretic peptide has demonstrated efficacy in reducing BBB damage and inhibiting neuronal apoptosis, and recombinant human brain natriuretic peptide treatment also reduces hippocampal inflammatory cytokine levels by suppressing the toll-like receptor 4/NF-κB pathway, resulting in improved 14-day survival rates and neurocognitive dysfunction in septic mice^204^. Systemic administration of dexmedetomidine, an α2-adrenoceptor agonist with sedative, anxiolytic, and analgesic properties, also was shown to attenuate the negative neurological effects of sepsis^205^. Further studies have revealed that the neuroprotective effects of dexmedetomidine are mediated by astrocyte α2A-adrenergic receptors^205^, ^206^. Therefore, ameliorating BBB injury is an important measure for managing the neurocognitive dysfunction caused by SAE.

### 4.2 Role of astrocytes in SAE

#### 4.2.1 Astrocytes and NLRP3/caspase-1

Bidirectional functional interaction occurs between astrocytes and NLRP3/caspase-1 signaling. First, in vitro studies have shown that exposure to LPS induces inflammatory vesicle activation and apoptosis in astrocytes^207^. In vivo, LPS treatment promotes caspase-1 immunoreactivity in astrocytes and triggers the release of the IL-1β and IL-18 pro-inflammatory cytokines^208^. Second, activation of NLRP3/caspase-1 inflammatory vesicles upregulates astrocyte IL-1β expression and effectively inhibits LPS-induced IL-1β expression by suppressing the expression of the astrocytic NLRP3/caspase-1 inflammasome^209^. In addition, treatment of astrocytes with lysophosphatidylcholine, a molecule associated with neurodegeneration and demyelination, induces the activation of the NLRP3 and NLRC4 inflammatory vesicles, which are central players in neuroinflammation and mediators of astrocyte proliferation^210^. Thus, astrocytes and NLRP3/caspase-1 signaling interact, each influencing the function of the other.

#### 4.2.2 Astrocytes and neuronal damage

Astrocytes play a crucial role in neuronal protection. As previously described, LPS treatment in rats promotes caspase-1 immunoreactivity in astrocytes and increases the release of IL-1β and IL-18 pro-inflammatory cytokines, leading to neuronal injury, which can be attenuated by dexmedetomidine. Research has suggested that dexmedetomidine protects glial cells and subsequently safeguards neurons by reducing apoptosis, which may preserve brain function and ultimately improve outcomes in patients with sepsis^162^. Furthermore, astrocyte-derived exosomes accumulate predominantly around neurons and were found to mitigate neuronal damage by inhibiting autophagy in vivo^211^, ^212^. Additionally, significant upregulation of 17β-estradiol 2 synthase aromatase expression occurs in astrocytes after brain injury. To investigate the precise role of astrocyte-derived estradiol 2 in the damaged brain, a GFAP promoter-driven aromatase knockout mouse model was established to deplete astrocyte-derived estradiol 2 in the brain, and the GFAP promoter-driven aromatase knockout mice showed intense neuronal damage, microglia activation, and neurocognitive dysfunction^213^. These findings underscore the multifaceted ways through which astrocytes protect neurons.

#### 4.2.3 Astrocytes and BBB damage

Astrocytes serve as crucial components of the BBB, actively contributing to its maintenance and regulation. Astrocytes express the glucagon-like peptide-1 receptor (GLP-1R), and activation of GLP-1R not only protects the BBB and reduces ischemia-induced neuroinflammation but also reduces the release of vascular endothelial growth factor A, matrix metalloproteinase 9 (MMP9), monocyte chemotactic protein-1 and chemokine ligand 1 (C-X-C motif). GLP-1R also increases the expression of tight junction proteins in brain endothelial cells, thereby improving BBB integrity^214^. Astrocytes can reduce neuron-derived C-C motif chemokine ligand 2 expression and protect BBB integrity by secreting fatty-acid-binding protein 7^215^. In mice deficient in pH-sensitive Na^+^/H^+^ exchanger 1 (NHE1), astrocytes release Wnt7a to activate the Wnt/β-catenin signaling pathway in cerebral vessels to protect BBB integrity^216^. The effect may be related to the control of caveolin-1 expression, vesicle abundance, and end-foot integrity in the neurovascular unit after activation of Wnt/β-catenin^217^. Astrocyte activation of pSTAT3 maintains cerebral vascular coverage through a process termed “endfoot plasticity,” which involves replacing nearby cells during the initial stages of astrocyte pedicle retraction, and the significant loss of endfoot coverage by astrocytes did not necessarily disrupt BBB integrity^218^.

However, under certain conditions, astrocytes can also contribute to BBB damage. Reactive astrocytes have been shown to disrupt BBB integrity in both in vivo and in vitro experiments^205^, ^219^. Oncostatin M secretion by reactive astrocytes induces BBB damage during neuroinflammation, which induces permeability and recruits T helper 17 cells through enhancing endothelial C-C motif chemokine ligand 20 secretion and integrin activation^220^. In response to inflammation, astrocytes contribute to BBB dysfunction through activation of STAT3 and increased expression of *SERPINA3*, which encodes α1-antichymotrypsin^221^. ApoE4 production by astrocytes leads to BBB leakage, increased MMP9, impaired tight junctions, and reduced vascular coverage of astrocyte terminals^222^. Under pathological conditions, N-myc downstream-regulated gene 2 binds directly to protein phosphatase and Mg^2+^/Mn^2+^-dependent 1A, increasing MMP9 expression in reactive astrocytes^223^. The activation of astrocytic p75 neurotrophin receptor further activates NF-κB and hypoxia inducible factor-1α signaling, upregulates MMP9 and vascular endothelial growth factor expression, and subsequently leads to post-ischemic tight junction degradation^224^. Therefore, astrocytes play an important role in both the onset of and recovery from BBB injury.

### 4.3 Summary

Indeed, the involvement of the NLRP3/caspase-1 complex in sepsis-induced hippocampal-dependent memory deficits underscores the critical role of inflammatory pathways in neurocognitive dysfunction. Inhibition of NLRP3 or caspase-1 not only reverses neurobehavioral abnormalities but also attenuates inflammatory responses, microglial activation, BBB disruption, and ultrastructural damage to brain tissue, protecting synaptic plasticity and thereby ameliorating neurocognitive dysfunction. The inflammatory response in astrocytes during SAE highlights the intricate interplay between astrocytes and inflammatory cascades. Regulation of astrocytic NLRP3/caspase-1 inflammatory vesicles can modulate the release of inflammatory factors, potentially influencing astrocyte proliferation and neuroinflammation. Additionally, reducing septic neuronal damage, possibly through astrocyte-mediated mechanisms like autophagy inhibition via released exosomes, holds promise for improving neurocognitive function in SAE. As astrocytes are important components of the BBB, they can resist BBB disruption. We have enumerated some of the receptors and pathways in astrocytes that positively regulate the BBB. However, under pathological conditions, astrocyte activation can lead to the formation of reactive astrocytes, which contribute to BBB damage and exacerbate neuroinflammation. In addition, dexmedetomidine can improve BBB leakage and neurocognitive dysfunction via the astrocyte α2A-adrenergic receptor. Overall, the emerging prominence of astrocytes in the context of SAE suggests that astrocyte inhibition may be a promising intervention to improve the neurocognitive dysfunction caused by SAE.

**Figure 5:**
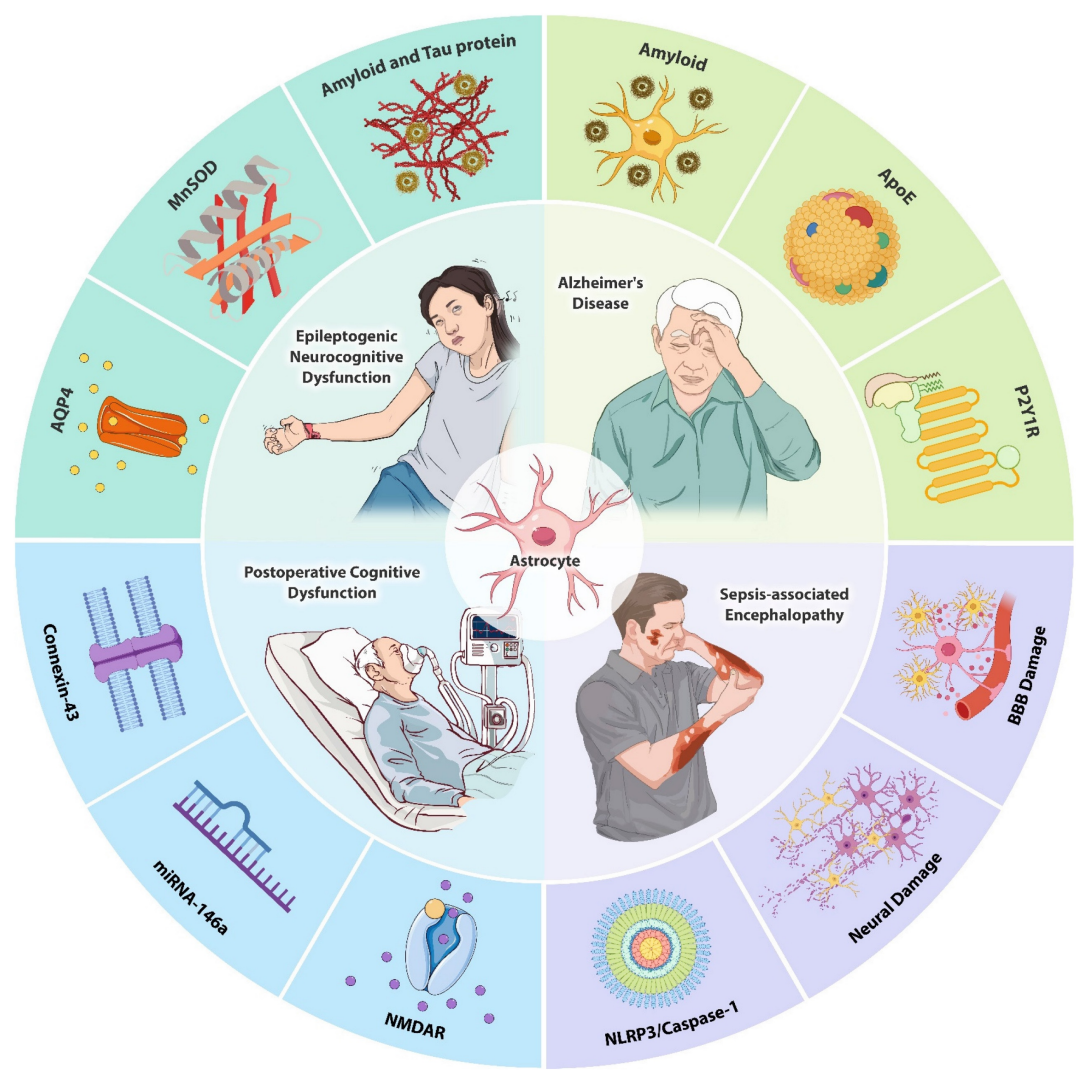
** Schematic overview of astrocyte involvement in neurocognitive dysfunction.** Under pathological conditions such as AD, epilepsy, POCD, and SAE, dysfunction of astrocytes leads to impaired neuronal signaling, increased oxidative stress, and altered synaptic activity, contributing to cognitive decline. The figure illustrates how astrocytes interact with neuroinflammatory pathways, modulate glutamate clearance, and regulate amyloid and tau protein deposition, all of which exacerbate neurocognitive dysfunction. Targeting astrocytic pathways may offer therapeutic opportunities for improving cognitive outcomes in various neurological disorders.

In summary, neurocognitive dysfunction can arise from a variety of conditions, including AD, epilepsy, exposure to surgery and anesthesia, and septicemic encephalopathy. These conditions impair cognitive function through different mechanisms, with astrocytes playing central and multifaceted roles in their pathogenesis. Astrocytes are involved in regulating synaptic activity, modulating inflammatory responses, and maintaining metabolic balance, all of which are critical for proper cognitive function (Fig. 5). Given their pivotal role in these processes, targeting astrocytes presents a promising therapeutic strategy for both preventing and treating neurocognitive dysfunction. By focusing on astrocytic pathways—such as modulating gliotransmitter release, controlling neuroinflammation, and enhancing synaptic plasticity—it may be possible to mitigate cognitive impairments across a range of neurological conditions. Thus, astrocytes offer viable targets for future research and clinical interventions aimed at improving cognitive outcomes in patients suffering from neurodegenerative diseases, post-operative complications, and other neurological disorders.

## Abbreviations

AD: Alzheimer's disease
POCD: postoperative cognitive dysfunction
SAE: sepsis-associated encephalopathy
JNK: c-Jun N-terminal kinase
mTOR: mammalian target of rapamycin
Aβ: amyloid-beta
MnSOD: manganese-containing superoxide dismutase
ROS: reactive oxygen species
NMDA: n-methyl-d-aspartate
AQP4: aquaporin 4
GLT-1: glutamate transporter 1
CNS: central nervous system
GFAP: glial fibrillary acidic protein
STAT3: transcription 3
JAK2: Janus kinase 2
BDNF: brain-derived neurotrophic factor
ACC: anterior cingulate cortex
NRG: neuregulin
APP: amyloid precursor protein
PKCα: protein kinase C alpha
TREM2: trigger receptor 2
P2Y1R: P2Y1 purinoreceptor
ApoE: apolipoprotein E
LTP: long-term potentiation
Cx43: connexin-43
GJ: gap junction
IRAK1: IL receptor associated kinase 1
miR-146a: MicroRNA-146a
NF-κB: nuclear factor kappa B
BTG2: B-cell translocation gene 2
NMDAR: N-methyl-D-aspartate receptor
TrkB: tropomyosin-related kinase receptor B
TrkB-FL: tropomyosin-related kinase receptor B-full-length
TrkB-T: tropomyosin-related kinase receptor B- truncated
IL: interleukin
NLRP3: Nucleotide-binding oligomerization domain (NOD)-like receptor protein 3
CLP: cecal ligation and puncture
LPS: lipopolysaccharide
Nrf2: nuclear factor-E2-related factor 2
HO-1: heme oxygenase-1
GLP-1R: glucagon-like peptide-1 receptor
MMP9: matrix metalloproteinase 9
AMPA: α-amino-3-hydroxy-5-methyl-4-isoxazole propionic acid
